# Combined periodontic-orthodonticendodontic interdisciplinary approach in the treatment of periodontally compromised tooth

**DOI:** 10.4103/0972-124X.70837

**Published:** 2010

**Authors:** D Deepa, D. S. Mehta, Viren K. Puri, Sadashiva Shetty

**Affiliations:** *Department of Periodontics, Subharti Dental College and Hospital, Meerut, Uttar Pradesh, India*; 1 *Department of Periodontology and Implantology India*; 2 *Department of Orthodontics, Bapuji Dental College and Hospital, Davanagere, Karnataka India*; 3 *Private Practice, Mumbai, India*

**Keywords:** Combined perio-ortho, interdisciplinary, orthodontic intrusion

## Abstract

Orthodontic treatment in adult patients is one of the most frequently encountered components involving multidisciplinary approaches. In the present report, a 28-year-old male patient was treated for localized chronic periodontitis with pocket formation, mobility, pathologic migration and malalignment of maxillary left lateral incisor tooth #22. The periodontal therapy included motivation, education and oral-hygiene instructions (O.H.I.), scaling and root planing and periodontal flap surgery. Subsequently on resolution of periodontal inflammation, orthodontic therapy was carried out using the orthodontic aligner for a period of 6 months. Post-treatment (3 years) results showed complete resolution of infrabony pocket with significant bone fill, reduced tooth mobility and complete alignment of the affected maxillary left lateral incisor, thus restoring the esthetics and function.

## INTRODUCTION

Pathologic migration of anterior teeth is an esthetic and functional problem that may be associated with advanced periodontal disease. The destruction of tooth-supporting structures is the most relevant factor associated with pathologic migration. Although some case reports have shown spontaneous repositioning of teeth following periodontal therapy alone, the treatment of severe cases of anterior spacing can be complex and time consuming and a multidisciplinary approach is often required including periodontal, orthodontic and restorative treatment.[1]

Orthodontic treatment in adult patients who have advanced periodontal disease can be performed by a team of clinicians, using a multidisciplinary approach to reestablish dentitions, both esthetically and functionally.[2] Aligning crowded or malposed maxillary or mandibular anterior teeth permits better access to adequately clean all surfaces of the teeth. This could be of tremendous advantage for patients who do not have the dexterity to adequately maintain their oral hygiene, or have susceptibility to periodontal bone loss.

A number of authors have tried to correct infrabony defects using orthodontic forces. Coronal tooth movement seems to be able to fill osseous defects if the alveolar bone follows the tooth in its displacement.[3] Clinical and istological studies indicate that new attachment is possible in association with orthodontic intrusion of teeth.[4] However, it seems possible to eliminate the defects by moving teeth with a reduced healthy periodontium into infrabony pockets, suggesting the possibility to investigate the presence of a new connective tissue attachment. Moreover, intrusive displacement has the potential to reestablish a healthy and well- functioning periodontium, with favorable psychological and esthetic results. After proper periodontal therapy, orthodontic treatment can positively improve both the alveolar bone and the soft periodontal tissues (Liu *et al*., 2008).[5]

This case report describes the benefits of integrating periodontal, orthodontic and endodontic therapies in case of chronic periodontitis that led to pathologic migration and extrusion of a maxillary left lateral incisor #22 with an osseous defect on its mesial aspect.

## CASE REPORT

**Figure 1:**
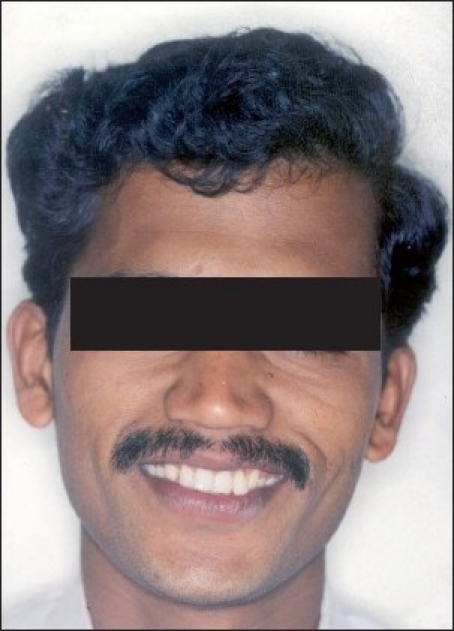
Pre-treatment extraoral photographs

A 28-year-old male patient presented to the Department of Periodontology and Implantology, Bapuji Dental College and Hospital, Davanagere, Karnataka, India, with a chief complaint of loose and extruded left upper front tooth which led to an irregular alignment. Thorough clinical examination revealed, the left lateral incisor had extruded and moved labially, with grade II mobility, with a deep periodontal pocket on its mesial surface. Complete radiographic evaluation (included panoramic and intraoral periapical radiographs) revealed a deep angular bony defect with periapical radiolucency. Intraoral clinical status assessment was made and the treatment was planned sequentially [Figures 1,2a–b and 3].

**Figure 2a:**
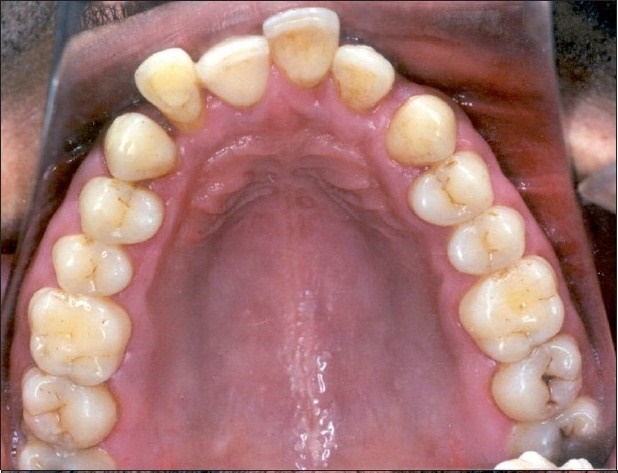
Pre-treatment intraoral photograph showing extruded maxillary lateral incisor #22

**Figure 2b:**
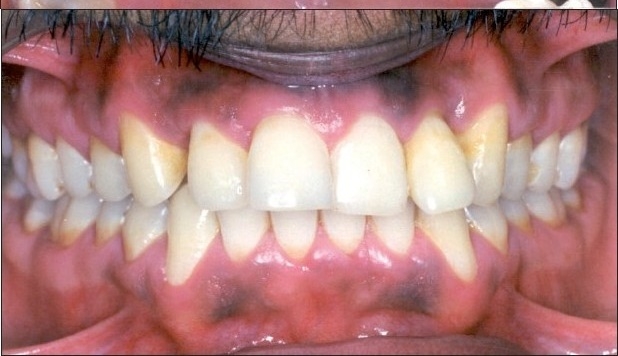
Pre-treatment intraoral photograph showing extruded #22

**Figure 3:**
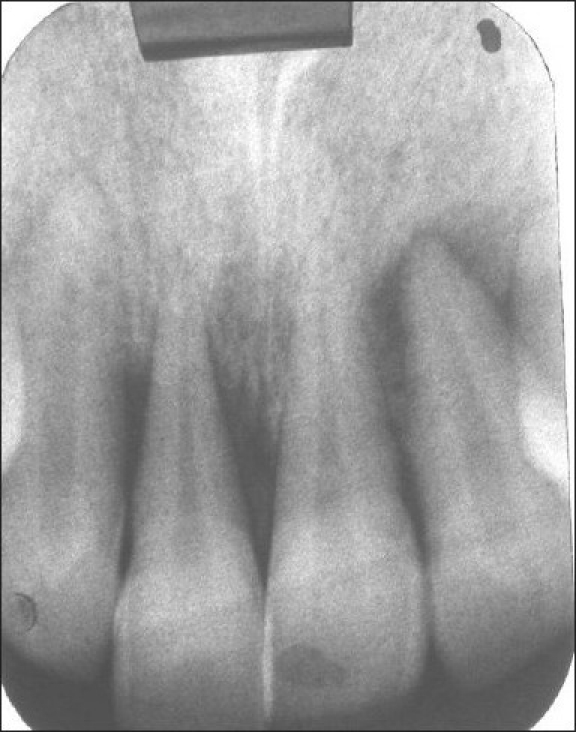
Pre-treatment radiograph showing osseous defect

The patient received supragingival scaling with oral hygiene instructions. Maxillary anterior teeth were splinted to improve stability and masticatory comfort. Following this, the maxillary left lateral incisor was treated with root-canal treatment to eliminate the periapical infection.

**Figure 4:**
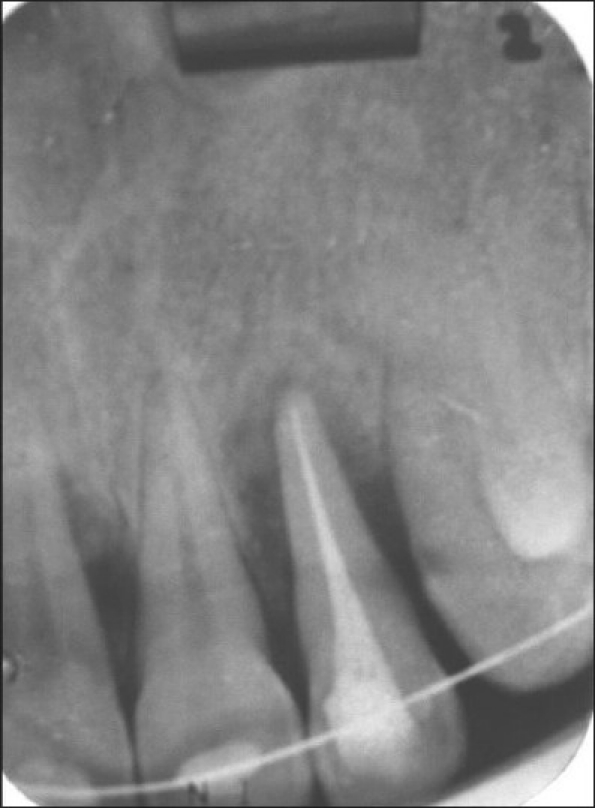
After periodontal flap surgery

**Figure 5:**
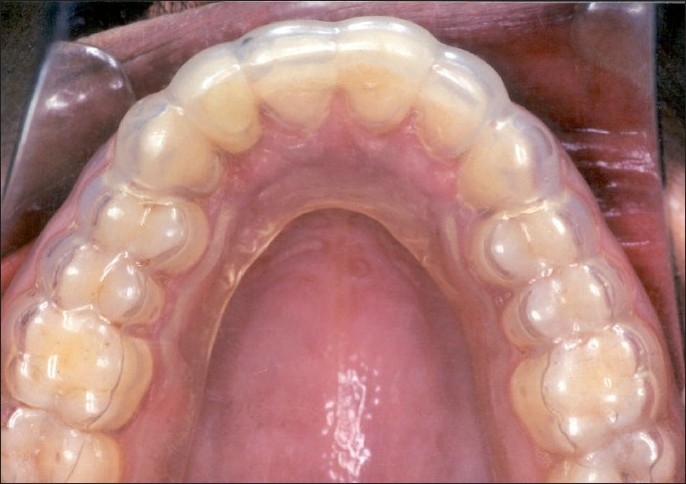
Placement of soft aligner used to realign lateral incisor #22

Six weeks later, the splint was removed and tooth mobility was assessed clinically. The affected tooth #22 showed only grade I mobility. Later, periodontal flap surgery was performed in the maxillary left lateral incisor area to completely eliminate the deep periodontal infection [Figure 4]. Intra-surgical observation confirmed the severity of the infrabony defect and loss of attachment. “Orthodontic aligner” was inserted 1 week after the surgical procedure for early stimulation of the connective tissue progenitor cells, necessary to foster regeneration [Figure 5]. The soft aligner ensures that the forces are not only light but also intermittent. This would allow regeneration of the periodontal tissue as tooth movement occurs. Fixed appliances were not used as the magnitude of tooth movement required was not very significant. Left lateral incisor was realigned in the maxillary arch, simultaneously moving the tooth apically and distally. The entire orthodontic treatment lasted for about 6 months.

### Appliance fabrication

An ideal diagnostic setup was done on upper working cast arranging the teeth in the desired position. A vacuum-formed tooth positioner was fabricated using the Biostar™ machine. A 1.5-mm Bioplast™ sheet was adapted over the upper model. Posteriorly, the occlusal surfaces of the molars and the premolars were relieved by cutting off the material. This ensured that the appliance remained thin enough for the patient’s comfort and did not ‘gag’ the mandible open. A 0.75-mm Imprelon™ was adapted over the entire arch for adequate rigidity and coverage of the posteriors. Step 1: Intrusion of #11 and #22 was carried out. Step 2: Minimal proximal slenderization was done from #12 to #22 with continued intrusion and alignment of #11 and #22. Step 3: Intrusion of #21 and #22 was carried out to level the anterior occlusal plane. Also derotation of #23 was done in order to close the space between #22 and #23.

### Retention

Begg retainer was modified to form a fitted labial bow anteriorly. Palatally, the acrylic was molded so as to act as a potential bite plane to prevent the supraeruption of lower anteriors. The patient was advised to wear it for a period of 12 months. During this period, the patient was recalled every 2 weeks, and oral prophylaxis was performed at regular intervals to control the inflammation in the interproximal region.

On post-treatment evaluation, a true intrusion of maxillary left lateral incisor #22 was observed without any evidence of extrusion of posterior teeth. It may be explained by the simultaneous application of low forces on the anterior teeth from soft Bioplast™ and the good adaptability and tight fit of orthodontic appliance over the posterior teeth resulting into intrusion of #22 without any evidence of posterior teeth extrusion, thus achieving an acceptable occlusion [Figure 6a–b]. Also, there was a normal gingiva showing no evidence of bleeding on probing and complete regaining of interdental papilla. Radiographic examination revealed significant fill of the infrabony defect [Figure 7]. Finally, the perio-ortho interdisciplinary approach resulted in the restoration of esthetics and function [Figure 8], thus improving self confidence of the patient.

**Figure 6a:**
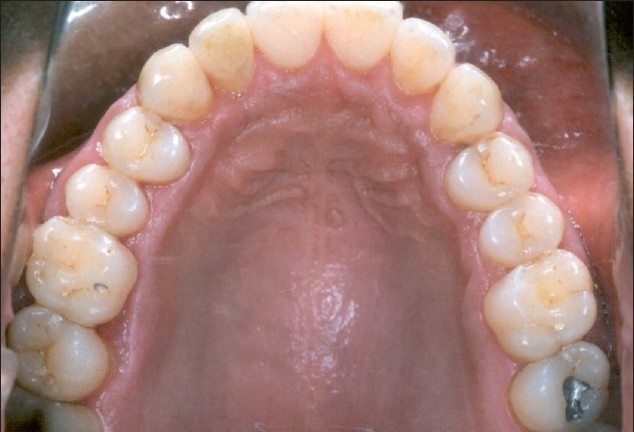
Post-treatment intraoral photographs showing complete alignment of maxillary anterior teeth

**Figure 6b:**
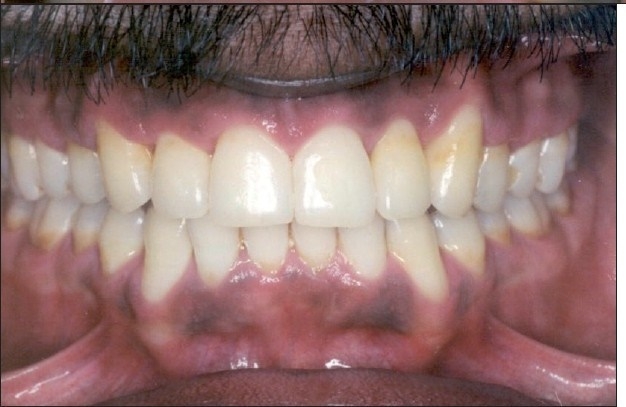
Post-treatment intraoral photographs showing complete alignment of maxillary anterior teeth and normal occlusion

**Figure 7:**
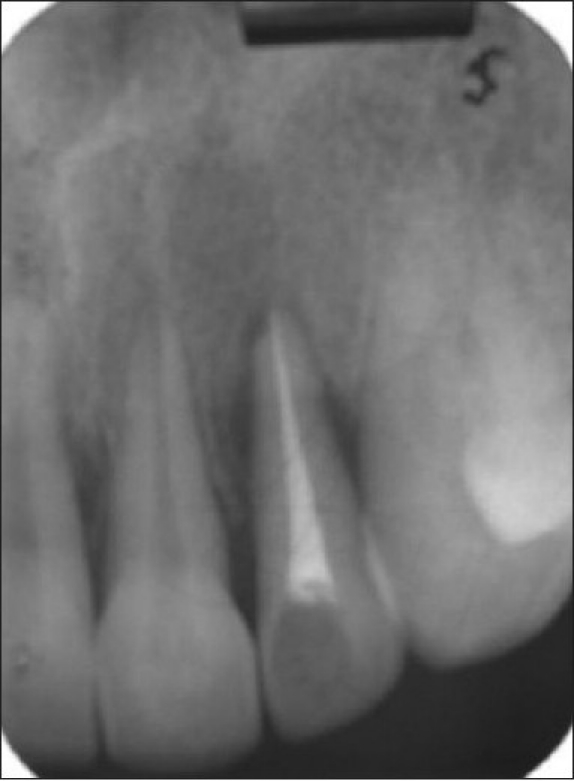
Post-treatment radiograph after periodontal flap surgery and intrusive orthodontic therapy

**Figure 8:**
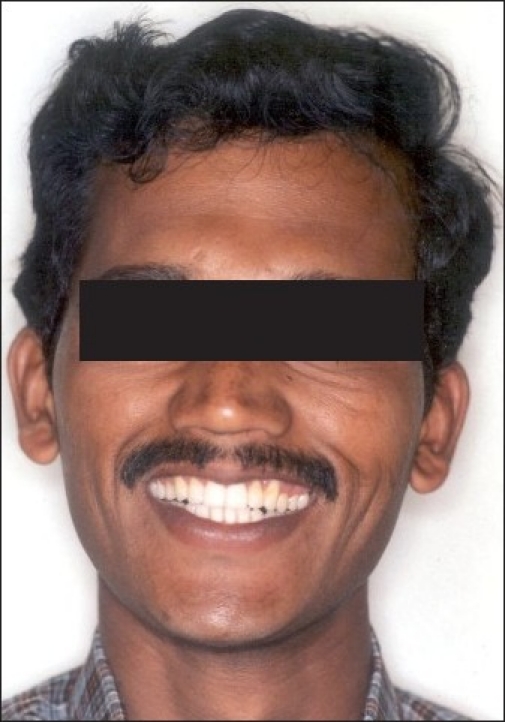
Post-treatment extraoral photographs

## DISCUSSION

The goal of periodontal therapy is to prevent disease progression and to regenerate the lost periodontal support. Over the years, open-flap surgery, graft materials and regenerative techniques have been used to achieve this goal in periodontal lesions with infrabony pockets.[6,7] Advanced periodontal disease may result in pathological migration (extrusion) and intraosseous defects. In such situations, orthodontic treatment may be a predictable therapeutic approach to realign migrated teeth, once periodontal therapy is completed.

Roberts and Chase[8] suggested that orthodontic tooth movement may enhance the mitotic activity of the periodontal ligament cells. Similarly, Melsen *et al*.[4] claimed that intrusive tooth movement may promote new attachment formation and seems to be a more effective and biologically conservative means to realign extruded tooth following periodontal therapy.[2,7] Early start of orthodontic movement (7 to 10 days after periodontal surgery) seems to be effective in determining the coronal shift of the soft tissues, which is an important concern from an esthetic point of view.

It has been suggested that stretching of the periodontal ligament fibers creates a natural barrier reducing the downgrowth of the epithelial cells. Orthodontic stimulation increases the turnover of the periodontal ligament cells and enhances the possibility of their repopulating the root surface.[9] However, these findings have to be confirmed by further investigation.

In some patients, periodontal therapy may cause spontaneous correction of pathologic migration. Spontaneous repositioning i n such cases can be attributed to the elimination of inflammation and the repair of transeptal and other gingival collagen fibers.[10] However, in the present case, there was no evidence of spontaneous repositioning of the teeth after periodontal treatment, probably because of the presence of severe bone loss around the involved incisor teeth.[11]

The present case report shows the effect of orthodontic tooth movement in a patient with a pathological migration (extrusion) of maxillary left lateral incisor (#22) resulting in esthetic and functional problems. The extruded lateral incisor was realigned and intruded, and the infrabony defect was undoubtedly healed without any bone grafting or guided tissue regeneration (GTR) procedures. In the present case, the malalignment was believed to be the result of pathologic migration, since the patient reported no previous crowding. Since esthetic improvement was required in the maxillary anterior region, it was necessary to use splinting to minimize the risk of secondary occlusal trauma that would result from excessive mobility of the teeth caused by extensive loss of supporting bone.[12] Space was created by reducing the proximal surface of the maxillary anterior teeth by proximal slicing for proper alignment, as well as by retracting the incisors to minimize tooth movement.[13]

Light orthodontic forces were applied to teeth with compromised bone support because they can move easily and larger forces can possibly negatively affect the periodontal membrane.[4,14] Periodontally compromised patients may have problems such as relapse during the retention stage, and therefore these patients require a long period of retention. Permanent retention is often part of the total treatment plan for these patients,[15,16] so that remineralization can be completed without the risk of relapse and to eliminate secondary occlusal trauma, thereby improving patient comfort.[17]

Reports on plaque control have stated that orthodontic tooth movement is possible without further bone or attachment loss when active treatment is started after inflammation is under control with periodontal therapy and with plaque control during the therapy period.[18,19] In this patient, a strict oral hygiene program was followed that included tooth brushing and rinsing at least twice a day as well as professional mechanical tooth cleaning every 2 weeks during the treatment period. This oral hygiene program and orthodontic treatment using light forces enabled favorable results to be accomplished without placing excessive stress on the periodontal tissues.

A combined orthodontic-periodontal approach can help modify the height of the papillae, an essential contribution to esthetic dentistry.[20] The patient maintained his natural dentition with a stable occlusion, acceptable masticatory function and pleasant esthetics, hence suggesting that orthodontic forces on healthy periodontium if kept within biologic limits do not cause periodontal breakdown but may enhance periodontal healing by their stimulatory effects on the periodontal ligament cells.

There are situations where orthodontic therapy can be performed to improve the prognosis of periodontally involved teeth. If performed meticulously, orthodontic therapy can certainly provide an excellent alternative to a clinician in maintaining the arch integrity and so the teeth hitherto doomed for extraction may be retained successfully, both to the satisfaction of the clinician and the patient. To achieve good results, it is essential to evaluate the individual morphology and topography of teeth and bone, the soft tissue characteristics and the biomechanical considerations.

## CONCLUSION

With increasing demand to improve their general quality of life, patients with compromised periodontal support also seek highly esthetic and functional improvements. Dental treatments of these patients are complex and challenging. However, interdisciplinary treatments that include periodontal treatment and orthodontic intrusion with endodontic therapy seem to be an effective method, provided both the biomechanical force system and the oral hygiene are kept under control, thus resulting in significant periodontal, esthetic and functional improvements.
